# OptiPrep Density Gradient Solutions for Nonmammalian Organelles

**DOI:** 10.1100/tsw.2002.839

**Published:** 2002-05-29

**Authors:** John M. Graham

**Affiliations:** School of Biomolecular Sciences, Liverpool John Moores University, UK

**Keywords:** gradient solutions, OptiPrep^TM^, iodixanol, density, osmolality, pH, yeast organelles, nonmammalian organelles

## Abstract

Any density gradient for the isolation of nonmammalian organelles should ideally only expose the sedimenting biological particles to an increasing concentration of the gradient solute. Thus they will experience only an increasing density and viscosity, other parameters such as osmolality, pH, ionic strength and the concentration of important additives (such as EDTA and DTT) should remain as close to constant as possible. This Protocol Article describes the strategies for the dilution of OptiPrep™ in order to prepare such solutions for organelles and membranes from nonmammalian sources such as yeast.

**METHOD TYPE:** extraction, isolation, purification and separation

**SUB METHOD TYPE:** centrifugation

## OSMOLALITY

Homogenates of yeast and plants and the solutions used to isolate organelles from these sources frequently contain either mannitol or sorbitol at concentrations (and osmolalities) significantly higher than those used for mammalian systems. The isolation of protoplasts or spheroplasts (from plants and yeast, respectively) is also carried out in such media in order to shrink the intact cell away from the cell coat. Media containing 400–600 mM mannitol or sorbitol are common but for yeast mitochondria concentrations as high as 0.8–1 M sorbitol are not unknown. Sucrose may also be used at up to 0.5 M.

The osmolality of iodixanol solutions is much lower than these values (a 50% (w/v) iodixanol is approx 195 mOsm); thus a special strategy has to be adopted to provide density solutions of the appropriate osmolality.

## pH

Because it is a solution in water with no additives, the pH of OptiPrep™ (4–6) is that of water. The actual pH is unimportant, because when OptiPrep™ is mixed with a standard ionic or organic buffer, the pH of the buffer is unaffected by the iodixanol; any changes in pH are the same as those that would occur during dilution of the buffer with water.

## HANDLING OptiPrep™

Prolonged exposure (several months) of iodixanol solutions to sunlight will cause a slow release of iodine; OptiPrep™ should therefore be stored away from strong sunlight. On standing, iodixanol may "settle out" of concentrated solutions; always shake the bottle of OptiPrep™ before use.

## PREPARATION OF DENSITY SOLUTIONS

The general strategy is to produce a high density Working Solution of the correct osmolality by diluting OptiPrep™ with a sorbitol (or mannitol) containing diluent and then diluting this solution with the normal homogenization medium (HM) or organelle suspension medium. Table 1 gives the properties of sorbitol solutions in 10 mM Tris-HCl, pH 7.4.

An example of the use of these diluents to produce gradient solutions of a constant osmolality (545–560 mOsm) is given below.

## METHOD

Prepare a Working Solution of 40% (w/v) iodixanol by diluting 4 vol of OptiPrep™ with 2 vol of 12.25% (w/v) sorbitol, 30 mM Tris-HCl, pH 7.4. This has a density of 1.225 g/ml.

Dilute the Working Solution with 8.75% (w/v) sorbitol, 10 mM Tris-HCl, pH 7.4 to provide gradient solutions of a suitable density (see Table 2). All of the solutions have an osmolality of approx 545 mOsm.

A Working Solution prepared using the 17.5% sorbitol diluent has a density of 1.231 g/ml and if this is then diluted with 12.25% sorbitol, 10 mM Tris-HCl, pH 7.4, all of the solutions will have an osmolality of approx 756 mOsm (see Table 3).

**Diluents containing mannitol** of the same concentration provide solutions of exactly the same density and osmolality.

**If sucrose** is used at the same **molar** concentrations (17.5, 12.25, and 8.75% sorbitol (mannitol) is equivalent to 0.96, 0.67, and 0.48 M, respectively) the solutions will have a similar, but not identical density and osmolality.

**TABLE 1**
**Density (ρ), Refractive Index (η), and Osmolality (Π) of Sorbitol Solutions in 10 mM Tris-HCl, pH 7.4**

| % (w/v) sorbitol | ρ (g/ml) | η | Π (mOsm) |
|---|---|---|---|
| 4.40 | 1.015 | 1.3390 | 265 |
| 8.75 | 1.029 | 1.3455 | 525 |
| 10.5 | 1.035 | 1.3480 | 657 |
| 12.25 | 1.041 | 1.3505 | 774 |
| 17.5 | 1.059 | 1.3580 | 1200 |

**TABLE 2**
**Density (ρ) and Refractive Index (η) of Iodixanol Solutions: Dilution of 40% Iodixanol Working Solution (WS), ρ = 1.225 g/ml with the 8.75% Sorbitol Diluent (D)**

| % w/v iodixanol | η | ρ (g/ml) | WS (ml) | D (ml) |
|---|---|---|---|---|
| 10.00 | 1.3593 | 1.078 | 2.50 | 7.50 |
| 12.00 | 1.3622 | 1.088 | 3.00 | 7.00 |
| 14.00 | 1.3650 | 1.098 | 3.50 | 6.50 |
| 16.00 | 1.3679 | 1.108 | 4.00 | 6.00 |
| 18.00 | 1.3707 | 1.117 | 4.50 | 5.50 |
| 20.00 | 1.3736 | 1.127 | 5.00 | 5.00 |
| 22.00 | 1.3764 | 1.137 | 5.50 | 4.50 |
| 24.00 | 1.3792 | 1.147 | 6.00 | 4.00 |
| 26.00 | 1.3821 | 1.157 | 6.50 | 3.50 |
| 28.00 | 1.3849 | 1.166 | 7.00 | 3.00 |
| 30.00 | 1.3878 | 1.176 | 7.50 | 2.50 |
| 32.00 | 1.3906 | 1.186 | 8.00 | 2.00 |
| 34.00 | 1.3935 | 1.196 | 8.50 | 1.50 |
| 36.00 | 1.3963 | 1.205 | 9.00 | 1.00 |
| 38.00 | 1.3992 | 1.215 | 9.50 | 0.50 |
| 40.00 | 1.4020 | 1.225 | 10.00 | 0.00 |

*Note*: Refractive index and density are related by the following simple equation: $\rho = 3.4394\eta - 3.5970$.

**TABLE 3**
**Density (ρ ) and Refractive Index (η ) of Iodixanol Solutions: Dilution of 40% Iodixanol Working Solution, ρ = 1.231 g/ml (WS) with the 12.25% Sorbitol Diluent (D)**

| % w/v iodixanol | η | ρ (g/ml) | WS (ml) | D (ml) |
|---|---|---|---|---|
| 10.00 | 1.3637 | 1.088 | 2.50 | 7.50 |
| 12.00 | 1.3663 | 1.098 | 3.00 | 7.00 |
| 14.00 | 1.3689 | 1.108 | 3.50 | 6.50 |
| 16.00 | 1.3716 | 1.117 | 4.00 | 6.00 |
| 18.00 | 1.3742 | 1.127 | 4.50 | 5.50 |
| 20.00 | 1.3769 | 1.136 | 5.00 | 5.00 |
| 22.00 | 1.3795 | 1.146 | 5.50 | 4.50 |
| 24.00 | 1.3821 | 1.155 | 6.00 | 4.00 |
| 26.00 | 1.3848 | 1.165 | 6.50 | 3.50 |
| 28.00 | 1.3874 | 1.174 | 7.00 | 3.00 |
| 30.00 | 1.3900 | 1.184 | 7.50 | 2.50 |
| 32.00 | 1.3927 | 1.193 | 8.00 | 2.00 |
| 34.00 | 1.3953 | 1.203 | 8.50 | 1.50 |
| 36.00 | 1.3979 | 1.212 | 9.00 | 1.00 |
| 38.00 | 1.4006 | 1.222 | 9.50 | 0.50 |
| 40.00 | 1.4032 | 1.231 | 10.00 | 0.00 |

*Note:* Refractive index and density are related by the following simple equation: $\rho = 3.6053\eta - 3.8280$.

## OTHER OSMOTIC BALANCERS

If the density of a diluent is known then the density of any iodixanol solution produced by mixing the diluent with OptiPrep™, an OptiPrep™ Stock, or a Working Solution can be calculated from Eq. 1.

$$D = \frac{Vd + V_1 d_1}{V + V_1} \tag{1}$$

$D$ = Density of mixture; $V$ = volume of OptiPrep™; $d$ = density of OptiPrep™; $V_1$ = volume of diluent; $d_1$ = density of diluent.

## CONCENTRATION OF BUFFER AND OTHER ADDITIVES IN THE GRADIENT

It may be important to maintain constant low concentrations (1–5 mM) of some additives such as EDTA or DTT in the gradient. In which case these should be added to the OptiPrep™ diluent at

3× the required gradient concentration when the Working Solution (WS) is prepared. If these additives are also included in the WS diluent (at their required concentration) then present in their concentration in all the density gradient solutions will be constant.

## ACKNOWLEDGEMENTS

The author and TheScientificWorld wish to thank Axis-Shield PoC, AS, Oslo, Norway for their kind permission to adapt OptiPrep™ Application Sheet S2 in the preparation of this Protocol Article.
